# The vascularized fascia lata free flap: an anatomical study and clinical considerations

**DOI:** 10.1007/s00405-020-05861-8

**Published:** 2020-02-25

**Authors:** Stefan Janik, Lena Hirtler, Hannes Traxler, Wolfgang J. Weninger, Rudolf Seemann, Boban M. Erovic

**Affiliations:** 1 Department of Otorhinolaryngology, Head and Neck Surgery, Medical University of Vienna, Vienna, Austria; 2 Center of Anatomy and Cell Biology, Medical University of Vienna, Vienna, Austria; 3 Institute of Head and Neck Diseases, Evangelical Hospital Vienna, Hans-Sachs Gasse 10-12, 1180 Vienna, Austria

**Keywords:** Vascular anatomy fascia lata, Fascia lata free flap, Fascia only ALT flap, Fascia lata

## Abstract

**Purpose:**

Fascia lata (FL) is often used as fascial component of the anterolateral thigh (ALT) flap in head and neck reconstruction. No distinct data exist on whether the FL has its own reliable vascular supply and whether the fascia alone can be harvested as a fascia lata free flap.

**Methods:**

We dissected 25 thighs of 15 cadavers. The lateral circumflex femoral artery (LCFA) was identified, and the size of stained fascia and skin were measured after injection of methylene blue into the descending branch (DB). Finally, topography of fascial vessels was determined.

**Results:**

Staining of fascia and skin paddle was found in all 25 cases. Ascending skin perforators of the DB of the LCFA gave off branches for supply of the FL enabling harvest of a fascia lata free flap. Septo- or musculocutaneous perforators pierced FL and entered skin within the proximal 38.6–60% of the thigh. The mean length and width of stained FL was 15.8 ± 4.1 cm and 8.7 ± 2.0 cm, respectively, and size of stained FL ranged from 40.0 to 336.0 cm^2^. In 20 cases (80%), skin paddles were 2.4 times larger on average compared to corresponding FL.

**Conclusion:**

We could demonstrate that the FL receives its own vascular supply from perforators of the DB originating from the LCFA. Hence, harvest of a fascia lata free flap is possible, reliable, and the size of the fascia is suitable for reconstruction of small and large defects of the head and neck.

## Introduction

The anterolateral thigh (ALT) flap was first described in 1984 by Song et al. [1] as fasciocutaneous free flap based on septo- or musculocutaneous perforators of the descending branch (DB) of the lateral circumflex femoral artery (LCFA) [2, 3]. The free ALT flap represents a highly versatile soft-tissue flap that can be utilized in various clinical situations [2]. Due to its low donor-site morbidity, ease of harvest, and versatility, the ALT has become the workhouse flap for soft-tissue reconstructions from head to toe [4].

The ALT flap is mainly harvested as fasciocutaneous free flap incorporating fascia lata (FL), overlying adipose tissue and skin, or as myocutaneous flap with additional muscular component to increase bulk [5–7]. The FL as fascial component of the ALT flap is preferred particularly for tendon reconstruction of lower and upper limbs [8, 9]. In addition, the FL is routinely used as non-vascularized graft in anterior skull base surgery or rhinology to cover small skull base defects.

The work of Stecco et al. [10] on complex vascular network and innervation of fascia sparked our interest in the possible vascular supply of the FL and whether the FL could be harvested as free fascia lata flap. In 1989, Koshima et al. had already discussed the ability to use the ALT flap as a free vascularized fascial flap, without skin or muscle, for reconstruction of dural or abdominal muscle defects [11].

Although numerous studies have been published on the clinical use of the ALT flap as well as on different harvesting techniques, there are a few works on the usage of a free vascularized FL flap so far. The size of possible free fascia lata flaps as well as their vascular supply is largely unknown. Therefore, we conducted an anatomical study to evaluate the size and location of perfused FL in correlation to corresponding skin paddles, and to describe the vascular supply of FL that may serve as basis for further clinical studies.

## Materials and methods

### Anatomical study

The anatomical part of the study was performed at the Center of Anatomy and Cell Biology of the Medical University of Vienna. All procedures performed in the study were in accordance with the ethical standards of the ethics committee of the Medical University of Vienna (1505/2015). Thirty paired thighs (*n* = 30) were dissected from 15 fresh specimens (8 female and 7 male). Five specimens were excluded from the analysis due to massive atherosclerosis.

## Dissection

Specimens were dissected in the dorsal decubitus position. To identify the LCFA, we initially performed a 10 cm skin incision in the groin penetrating down to the FL. The skin and subcutaneous tissue was raised within this plane until the great saphenous vein was found. The latter was dissected proximally until the saphenous hiatus was identified. Thereafter, we slightly incised the FL to identify the deep femoral artery. The LCFA represents the first branch of the deep femoral artery that gives off the ascending, descending, and transverse branches (Fig. 2a, b). Finally, the ascending (AB) and transverse branches (TB) were clamped and ligated, while the descending branch (DB) was cannulated with a 20G syringe and 40 ml methylene blue was injected.

### Assessment of staining

We separately evaluated the size and area of stained fascia and corresponding skin paddle, for which we used a commonly available 1.0 mm ruler and gauged the longest vertical (length) and horizontal (width) diameter. The intersection between those diameters was assessed as midpoint of the stained skin island. In addition, we used a coordinate system consisting of anatomic landmarks [anterior superior iliac spine (ASIS) and upper border of the patella] to describe and analyze location and extent of stained fascia and skin area. A drawn line running from the ASIS to the upper border of the patella was used as reference line, and as surrogate marker for the length of the thigh. Having evaluated size and location of the skin paddle, we performed a hockey stick-like skin incision starting in the groin and running along the medial side of the thigh in curvilinear fashion above the upper border of the patella. The incision penetrated to deep fascia, and after removing subcutaneous tissue, we could assess the stained fascial paddles.

### Statistical methods

SPSS (version 26; IBM SPSS Inc., Chicago, IL, USA) was used for statistical analysis of data. Unless otherwise specified, metric data are represented as mean ± standard deviation in the result section. Kolmogorov–Smirnov test was performed to test for normal distribution of metric variables. An unpaired Student’s *T* Test and Mann–Whitney *U* test were used to compare means of normally and non-normally distributed variables. A Chi-square test was applied to assess nominal variables and a paired student’s *T* test was used to analyze two dependent groups. Pearson correlation (*r*) was performed to analyze linear relationships between two numerical measurements. All tests were two-sided and *p* values below 0.05 were considered statistically significant.

## Results

### Anatomical study

#### Skin paddles

**Fig. 1:**
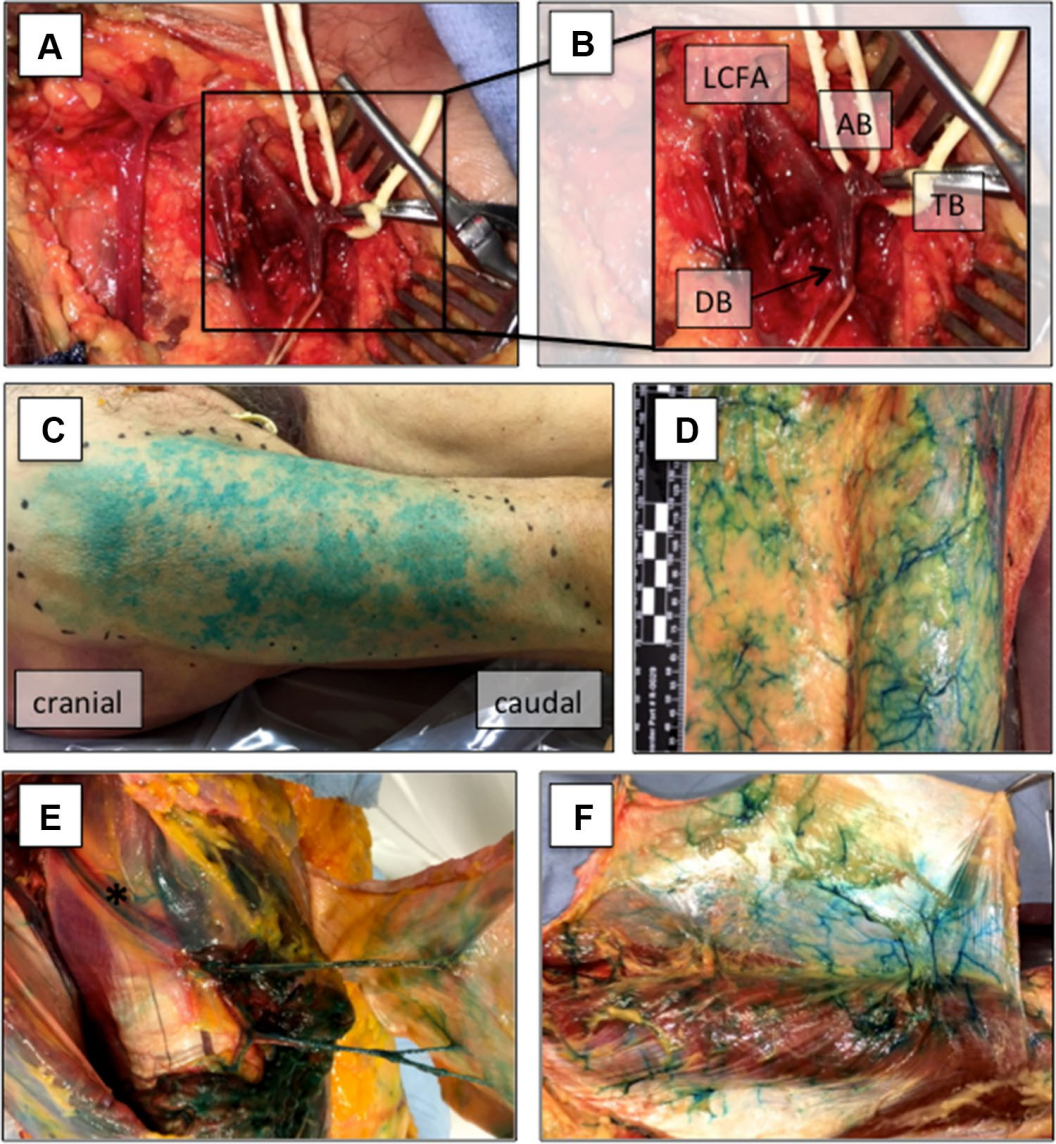
Vascular supply of the fascia lata. The lateral circumflex femoral artery (LCFA), the descending (DB), ascending (AB), and transverse branches (TB) were identified first (**a**, **b**). Thereafter, the DB was cannulated and 40 ml methylene blue were injected (not shown). After we assessed the maximal length and width of stained skin paddles (**c**), we elevated the adipocutaneous tissue suprafascially from medial to lateral (**d**). To protect the peri-fascial blood supply, we preserved some adherent adipose tissue. The fascia lata was supplied by septocutaneous (**e**) or musculocutaneous (**f**) perforators. Asterisk (*) marks the DB of the LCFA within the intermuscular septum after retraction of the rectus femoris muscle

Skin islands of the DB of the LCFA were successfully stained in all 25 cases (100%; Fig. 1c). The mean length and width of dyed skin islands was 19.7 ± 7.6 cm (range 5.0–35.0 cm) and 12.0 ± 3.9 cm (range 4.0–20.0 cm), respectively. The mean area of stained skin paddles was 249.3 ± 130.2 cm^2^ with a range of 20.0–528.0 cm^2^ (Table 1). Size of skin paddles did not significantly differ between males and females (240.5 ± 128.7 cm^2^ vs. 257.4 ± 136.3 cm^2^; *p* = 0.754), left compared to right thighs (238.5 ± 123.9 cm^2^ vs. 260.9 ± 141.2 cm^2^; *p* = 0.676), or between paired specimens (left vs. right: 262.0 ± 113.5 cm^2^ vs. 269.7 ± 144.7 cm^2^; *p* = 0.827).

**Table 1:** Stained fascia lata and corresponding skin paddle

Case	Sex	Paired	Side	Fascia lata		Skin paddle		Length of thigh	Midpoint of flap	
				Length	Width	Length	Width			
				cm	cm	cm	cm	cm	cm	%
1	F	Yes	Right	13	8	16	8.5	48	24.5	51
2	F	Yes	Left	15.5	8	13	8	48.5	24	49.5
3	F	No	Left	10	9.5	5	4	44	17	38.6
4	M	Yes	Left	12	9	20	14.5	46	23	50
5	M	Yes	Right	16	9	7	6.5	45	23	51.1
6	F	Yes	Right	14	8	17.5	15.5	45	27	60
7	F	Yes	Left	14	8	18	12.5	45	27	60
8	M	Yes	Right	24	14	33	16	48	24	50
9	M	Yes	Left	24	11	30	10.5	47	26	55.3
10	F	Yes	Left	18	8	22	10	44	22	50
11	F	Yes	Right	16	8	18	11	44	22	50
12	M	Yes	Right	21	6.5	35	8	60.5	35.5	58.7
13	M	Yes	Left	19.5	7.5	32.5	10	60.5	35.5	58.7
14	F	Yes	Right	15	10	26	16	49.5	24	48.5
15	F	Yes	Left	22	9.5	22	17.5	50	22.5	45
16	M	Yes	Right	11	6.5	19	16	49,5	26	52.5
17	M	Yes	Left	16.5	10.5	16	13	49	27	55.1
18	M	No	Right	14.5	9.5	15	11	52	26.5	51
19	M	Yes	Left	20	12.5	14	8	44	24	54.5
20	M	Yes	Right	15	10	10.5	11	44	24	54.5
21	F	Yes	Left	18	9	27	18	45	23	51.1
22	F	Yes	Right	15	8.5	21	20	44	17	38.6
23	F	Yes	Right	10	7	21	12	48.5	29	59.8
24	F	Yes	Left	10	6	17	12.5	48.5	26.5	54.6
25	M	No	Left	12	4	18	11	45	21	46.7

The maximum length (cm) and width (cm) of fascia lata and stained skin paddles are indicated. A drawn line between the anterior superior iliac spine (ASIS) and the upper border of the patella was used as surrogate marker for the length of the thigh. The midpoint of stained skin paddle and perforators was indicated with reference to the ASIS

*M* male, *F* female

#### Flap location

**Fig. 2:**
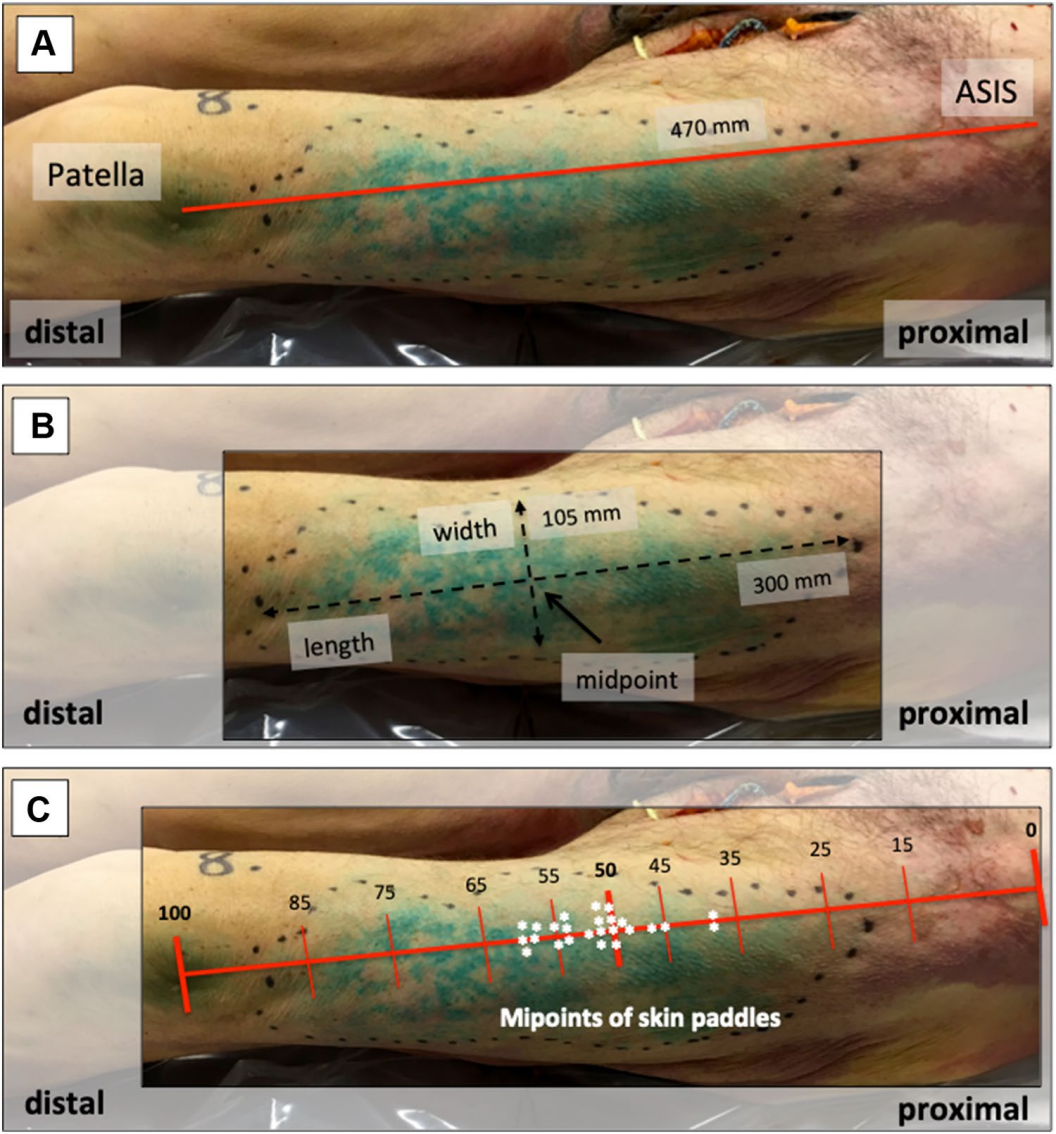
Location of perforators. The anterior superior iliac spine (ASIS) and the upper border of the patella were used as anatomic landmarks. A drawn line between those landmarks was used as reference line and as surrogate marker to assess the length of the thigh (100%), the location of skin paddles and entrance of perforators (**a**). The intersection between the maximal length and width of stained skin islands was used as midpoint indicating entrance of perforators (**b**). All midpoints were located within the proximal 35–60% of the thigh (**c**)

We used a drawn line between the ASIS (proximal) and the upper border of the patella (distal) as indicator for the length of the thigh. The mean length of the entire thigh, representing 100%, was 47.8 ± 4.5 cm, ranging from 44.0 to 60.5 cm. The highest density of skin perfusion, indicating the entrance of cutaneous perforators, was found within the proximal 35–45%, 45–55%, and 55–60% in 2 (8.0%), 16 (64.0%), and 7 (28.0%) cases, respectively (Fig. 2).

#### Stained fascia lata

After assessing stained skin islands, we removed the cutaneous and subcutaneous tissue of the thigh to evaluate the pattern and size of stained FL. We observed that ascending cutaneous perforators pierced FL and gave off separate branches for FL supply. Importantly, perforators entered skin perpendicularly to FL within the previously determined skin islands.

We detected stained FL in all 25 cases (100%), and after suprafascial removal of subcutaneous tissue (Fig. 1d), the mean length and width of stained FL was 15.8 ± 4.1 cm and 8.7 ± 2.0 cm, respectively. Assuming rectangular paddles, the approximate area of stained FL was 142.5 ± 65.6 cm^2^ (range 48.0–336.0 cm^2^). This was significantly smaller compared to corresponding skin paddles (249.3 ± 130.2 cm^2^; *p* = 0.002; Table 1). However, size of stained FL did not significantly differ in males and females (*p* = 0.122) or right compared to left thighs (*p* = 0.800).

#### Size of stained fascia compared to skin paddles

In 20 cases (80%), the mean size of skin islands was 2.4 times larger compared to stained FL (range 1.2–4.3-fold). In the remaining 5 cases (20%), however, stained FL was 1.2–4.7 times larger (mean 2.5-fold) than skin paddles. Although there was a trend towards larger stained FL areas in specimens with larger skin paddles, differences failed to reach statistical significance (*r*: 0.383; *p* = 0.059).

#### Harvest of a fascia lata free flap: clinical considerations

After successfully demonstrating that FL has a reliable vascular supply, we harvested a fascia lata free flap. First, the skin incision was marked incorporating the midpoint of the stained skin paddle. In clinical practice, a handheld Doppler has to be used to identify the main skin perforators. Next, we identified the ascending perforators and determined the exact position where they pierced FL. Thereafter, FL was incised incorporating the stained fascial paddle, and the fascia lata free flap was further elevated subfascially to identify septo- or musculocutaneous perforators (Fig. 1e, f). These perforators were dissected proximally towards the LCFA. We typically found 2–3 main ascending perforators of the DB of the LCFA supplying the fasciocutaneous tissue (Fig. 1e, f). After complete dissection of the supplying vascular pedicle, the overlying subcutaneous and cutaneous tissue was retracted and removed from FL. Finally, a fascia lata free flap can be harvested ranging from 4.0 × 10.0 cm to 14.0 × 24.0 cm (Table 1) in size.

## Discussion

We could demonstrate that the ascending skin perforators of the DB gave off separate branches for fascia lata supply. Similarly, Wavreille et al. [12] found that the use of a free fascial lateral arm flap was based on a rich vascular supply with small arteries and an abundant venous system that is located strictly between the deep and superficial aspects of fascia.

In our series, the mean area of ALT skin islands was 249.3 ± 130.2 cm^2^ ranging up to almost 525.0 cm^2^. Other works found even larger skin islands with mean sizes of 341 cm^2^ and 365 cm^2^, respectively, based on one single perforator [13, 14]. In the majority of our cases, skin paddles were approximately 2.4 times larger than the stained fascia itself. The heterogeneity of our data—with stained fascial paddles ranging from 4.7 times larger to 4.3 times smaller in size compared to skin paddles—may be caused, at least partially, by the method that we used. Although the performance of cannulation and injection of dye is well established [15–17], we cannot rule out that anatomic variations or additional branches were stained after application of 40 ml methylene blue in some cases.

Nonetheless, our observation is of particular interest, since we could show that perforators of the DB supply first the fascia and second the overlying subcutaneous tissue and skin. According to our data, fascia lata free flaps with at least 4.0 × 10.0 cm (40 cm^2^) in dimension to almost 14.0 × 24.0 cm (336 cm^2^) can be harvested.

Some surgeons might be concerned about skin perfusion when large fascial flaps with corresponding perforators are harvested. Saint Cyr M. and coworkers, however, demonstrated that perforators of the LCFA system are linked by interconnections particularly at the suprafascial level and the adipose layer of the deep fascia. This dense suprafascial and subcutaneous vascular network enables the harvest of ALT flaps with cutaneous skin paddles of up to 630 cm^2^ [14] as well as harvest of fascial flaps while maintaining skin perfusion.

In clinical routine, a handheld Doppler is used to identify skin perforators of the ALT flap [2]. These perforators are typically located in the middle third or proximal half of a reference line drawn between the ASIS to the upper border of the patella [2, 18]. In accordance to this, all midpoints of stained skin paddles of our specimens, indicating the entrance of ascending skin perforators, were located in the middle third of this reference line.

In the past 30 years, the ALT flap has become a workhouse flap for soft-tissue reconstruction and numerous studies with large patient numbers extensively discussed the possible applications, different harvesting techniques, and anatomic variations of free ALT flaps [2, 5–7, 19]. However, only 5 articles described the use of an ALT fascia only free flap in 38 different cases [20–24]. Free fascia lata flaps were successfully described with a 97.4% free flap survival rate for recreation of mucosal lining after total or subtotal rhinectomy (*n* = 5) [21], reconstruction of palatomaxillary defects (*n* = 14) [22], extremity (*n* = 5) [20], and upper limb reconstruction (*n* = 6) [23], as well as for coverage of mandibular bone after osteoradionecrosis secondary to radiation therapy (*n* = 8) [24].

Fox et al. [20] found improved esthetic outcomes of fascia only ALT flaps regarding contour and color match compared to myocutaneous and fasciocutaneous ALT flaps in extremity reconstruction. Moreover, Seth et al. [21] indicated that the low donor-site morbidity, and the thinness and pliability of the flaps are optimal for inner lining of the airway contour of the nose. A thin, pliable, vascularized fascia lata free flap may also be useful for microtia reconstruction to cover either artificial material (e.g., MEDPOR®) or autogenous costal cartilage, to prevent wound infections, improve wound healing, and as additional resistant layer against mechanical stress. It has already been established that non-vascularized biological meshes made of the FL are more resistant to infections and are more easily integrated in recipients’ sites compared to artificial meshes [25].

Nonetheless, we are aware of the fact that the ALT fascia only free flap has of course restricted indications and applications in head and neck area compared to established free flaps, like the ALT or radial forearm free flap (RFFF). However, one may use the ALT fascia only free flap to overcome with potential drawbacks and limitations of the abovementioned flaps. First, the ALT fascia only free flap represents a thin, vascularized flap with no tissue bulk and, therefore, less or absent atrophy, and no need for secondary surgeries with thinning of the flap to improve contour compared to the ALT flap [20, 24]. Second, even large ALT fascia only free flaps can be harvested by a straight-line donor incision, while large fasciocutaneous ALT flaps often require split-thickness skin grafts for closure [20, 26]. Third, the ALT fascia only free flap allows harvest of larger flap sizes and has no hair-bearing tissue, which is particularly important for mucosal reconstruction, and low donor-site morbidity compared to the RFFF [21].

However, it is noteworthy to mention that ALT perforators—consequently also perforators of the fascia lata free flap—are absent in 0.7–11.2% of cases [27]. Several published reports described converting to a tensor fascia lata (TFL) flap in the absence of perforators [28, 29]. Recently, Powers et al. [30] successfully described the perforator anatomy of the TFL compared to the ALT flap by performing a high-resolution computed tomography angiography (CTA). They reported of a significantly higher perforator density of the TFL compared to the ALT flap. These findings were further reflected by the work of Hubmer et al. [31] demonstrating the constant anatomy, especially of septocutaneous perforators arising from the AB of the LCFA between the tensor fascia lata and the medial gluteal muscle, and, therefore, the reliability of the TFL flap. Hence, the TFL flap represents an attractive alternative from a similar regional donor site in cases with inappropriate or even absent ALT perforators.

Although our study provides anatomical data on the harvest of a free fascial flap for the first time, it has several limitations. First, the value of injection studies with methylene blue and their clinical impact is questionable. The size of skin paddles and location of skin perforators, however, are in line with the current literature, thus supporting the accuracy of our data. The lack of clinical cases and additional techniques to describe the vascular network of the FL in greater detail represent additional drawbacks of our work. The feasibility and reliability of the flap, on the other hand, is underscored by the literature reports of 38 successful cases with almost 100% flap survival in 5 independent centers.

With regards to the harvest of a fascia lata free flap, preservation of skin and subcutaneous tissue aid in the manipulation of the flap during harvest and thus helps to avoid damage of the vascular plexus surrounding the FL and perforators [22]. A free fascia lata flap has to be incised and harvested in the required size incorporating the perforator, which is traced and dissected to the source of the pedicle creating perforators ranging from 8 to 16 cm in length with a diameter of more than 2 mm [3]. We recommend to preserve some adherent adipose tissue to protect the vascular supply rather than to completely uncover the fascial surface. This is because the fascial blood supply seemed to be located more on the superficial than on the caudal surface of the fascia, and vascular interconnections mainly located at the suprafascial level of the fascia [14].

## Conclusion

We could demonstrate that the fascia lata has its own reliable vascular supply enabling harvest of a free fascia lata flap. We are aware of the fact that indications for a fascia lata flap are limited, but nonetheless, we believe that a free FL flap has its value, particularly where thin, pliable, hairless, and well-vascularized flaps are needed. Therefore, the possibility to harvest a fascia lata flap only further adds to the versatility of the ALT flap. However, additional studies are warranted to assess further potential applications and limitations of a free fascia lata flap.
